# Mutation of the glycine residue preceding the sixth tyrosine of the LAT adaptor severely alters T cell development and activation

**DOI:** 10.3389/fimmu.2022.1054920

**Published:** 2022-12-07

**Authors:** Mikel M. Arbulo-Echevarria, Inmaculada Vico-Barranco, Fanghui Zhang, Luis M. Fernandez-Aguilar, Martyna Chotomska, Isaac Narbona-Sánchez, Lichen Zhang, Bernard Malissen, Yinming Liang, Enrique Aguado

**Affiliations:** ^1^ Department of Biomedicine, Biotechnology and Public Health (Immunology), Universidad de Cádiz, Cádiz, Spain; ^2^ Institute of Biomedical Research Cadiz (INIBICA), Cádiz, Spain; ^3^ Centre d’Immunologie de Marseille-Luminy (CIML), Aix Marseille Université, INSERM, CNRS, Marseille, France; ^4^ Henan Key Laboratory for Immunology and Targeted Therapy, School of Laboratory Medicine, Xinxiang Medical University, Xinxiang, China; ^5^ Laboratory of Immunophenomics, School of Laboratory Medicine, Xinxiang Medical University, Xinxiang, China

**Keywords:** linker for activation of T cell, TCR (T cell receptor), T cell development, anergy, T cell memory, T cell

## Abstract

The LAT transmembrane adaptor is essential to transduce intracellular signals triggered by the TCR. Phosphorylation of its four C-terminal tyrosine residues (136, 175, 195, and 235 in mouse LAT) recruits several proteins resulting in the assembly of the LAT signalosome. Among those tyrosine residues, the one found at position 136 of mouse LAT plays a critical role for T cell development and activation. The kinetics of phosphorylation of this residue is delayed as compared to the three other C-terminal tyrosines due to a conserved glycine residue found at position 135. Mutation of this glycine into an aspartate residue (denoted LAT^G135D^) increased TCR signaling and altered antigen recognition in human Jurkat T cells and ex vivo mouse T cells. Here, using a strain of LAT^G135D^ knockin mice, we showed that the LAT^G135D^ mutation modifies thymic development, causing an increase in the percentage of CD4+CD8+ double-positive cells, and a reduction in the percentage of CD4+ and CD8+ single-positive cells. Interestingly, the LAT^G135D^ mutation alters thymic development even in a heterozygous state. In the periphery, the LAT^G135D^ mutation reduces the percentage of CD8+ T cells and results in a small increment of γδ T cells. Remarkably, the LAT^G135D^ mutation dramatically increases the percentage of central memory CD8+ T cells. Finally, analysis of the proliferation and activation of T lymphocytes shows increased responses of T cells from mutant mice. Altogether, our results reinforce the view that the residue preceding Tyr136 of LAT constitutes a crucial checkpoint in T cell development and activation.

## Introduction

The stochastic generation of T-cell antigen receptors (TCRs) allows the production of a wide repertoire of antigen receptors. However, due to the stochastic nature of this process, TCR recognizing self-antigens can be generated, which must be regulated by central or peripheral tolerance mechanisms. During T cell development, T cell precursors rearranging TCR genes must pass several checkpoints in which the quality of the assembled receptors is tested. Accordingly, T cells the TCR of which does not match self-peptide MHC (pMHC) products are eliminated together with those that could recognize self-pMHC products with a too high affinity (1).

The recognition of pMHC ligands by T cells generates a cascade of intracellular signals, and one of the earliest is the phosphorylation of ITAMs present in the intracellular domains of CD3 chains (2). Phosphorylation of ITAMs by the tyrosine kinase Lck generates binding sites for the recruitment of the ZAP-70 cytosolic tyrosine kinase, which in turn is activated by Lck-mediated phosphorylation. Activated ZAP-70 phosphorylates tyrosine residues in the adaptor protein LAT, which coordinates the assembly of a multiprotein signalosome linking the TCR-CD3 complex to the main intracellular downstream signaling pathways (3, 4). LAT has a critical function in T-cell signaling, and its activity is mainly channeled through the four C-terminal tyrosine residues found at positions 136, 175, 195, and 235 (mouse numbering) (5, 6).

Regulation of the intensity of intracellular signals delivered by the TCR is of crucial relevance for T cell development and activation. Interestingly, it has been demonstrated that the sixth tyrosine of LAT (Tyr136 in mice) plays a dual role in this complex signaling cascade. This is the only residue of LAT able to bind to PLC-γ1, and its mutation to Phe prevents PLC-γ1 activation and calcium concentration increase in response to TCR engagement. A LAT-knockin (LAT-KI) strain of mice in which Tyr136 was mutated to phenylalanine (LAT^Y136F^) displays a severe but partial block in T cell maturation, but later develop a polyclonal lymphoproliferative disorder involving CD4+ T cells producing massive amounts of T helper type 2 (TH2) cytokines (7, 8). These paradoxical phenotypes established for the first time that LAT is a crucial regulator of T cell homeostasis, a finding later confirmed using mice in which the LAT^Y136F^ mutation was only expressed in the periphery (9).

It has been shown that ZAP-70 preferentially phosphorylates the tyrosine residues that are found in the LAT and SLP-76 adaptors and are surrounded by negatively-charged residues (10). This mechanism precludes ZAP-70 from phosphorylating its own activation loop, thereby ensuring its dependence on Lck for activation. LAT tyrosine 136 of mouse LAT is preceded by an evolutionarily conserved non-charged glycine residue. This delayed the phosphorylation kinetics of LAT-Y136 as compared to the three tyrosine residues found at the C-terminus of LAT (11). More recently, it has been demonstrated that the slow phosphorylation of LAT-Y136 can be accelerated by substituting this glycine with aspartate or glutamate, leading to an increased sensitivity of T cells to weak agonists and self-peptides when assayed using transformed T cells and ex vivo T cells (12). In order to gain insight into the *in vivo* relevance of the phosphorylation kinetics of LAT-Y136, we generated a strain of knockin (KI) mice in which glycine 135 was mutated to an aspartate (LAT^G135D^). LAT^G135D^ mice showed altered thymic development, as well as abnormal peripheral T-cell populations among which CD8+ T cells showed the strongest phenotype. Interestingly, the LAT^G135D^ mutation was found to exhibit a dominant behavior, since heterozygous animals showed a phenotype intermediate between wild-type and homozygous mutant mice. Therefore, our *in vivo* and ex vivo results reinforce the view that the glycine residue preceding the Tyr136 of LAT constitutes a crucial checkpoint in T cell development and activation.

## Materials and methods

### Antibodies and reagents

The anti-CD3, -CD4, -CD5, -CD8, -CD24, -CD25, -CD44, -CD62L, -CD69, -CD73, -CCR7, FoxP3, and -FR4 fluorochrome-coupled antibodies, and Annexin V-APC reagent used for cytometry were from BioLegend (San Diego, San Diego, CA, USA). Antibodies specific for P-PLC-γ1 (Y783), LAT and β-Actin were from Cell Signaling Technologies (Danvers, MA, USA). Anti-P-Y132-LAT antibody was from Abcam (Cambridge, MA, USA). P-Erk and PLC-γ1 antibodies were from Santa Cruz Biotechnology (Heidelberg, Germany). Erk antibody was from BioLegend. CellTrace™ Violet and Dynabeads^®^ Mouse T-Activator CD3/CD28 reagents were from Invitrogen (ThermoFisher Scientific, Waltham, MA, USA). Biotin anti-mouse CD3ϵ and streptavidin were from BioLegend.

### Animals

Mice were maintained and used at the local Animal Supply Services (SEPA, University of Cadiz). Animal care and handling followed the guidelines of the European Union Council (2010/63/EU, 86/609/UE) on the use of laboratory animals. Experimental procedures were approved by the local Animal Care and Ethics Committee (University of Cadiz, Cadiz, Spain) and the Ministry of Agriculture, Fisheries and Rural Development (Junta de Andalucía, Spain).

### Generation and validation of knockin mouse expressing a *Lat* ^G135D^ allele

A chimeric single guide RNA (sgRNA) targeting the sequence 5’-GATGAAGACGACTATCCCAA *CGG-*3’ (the protospacer adjacent motif (PAM) is shown in italics) found in exon 7 of the *Lat* gene was synthesized. A single-stranded deoxynucleotide (ssDNA) homology-directed repair (HDR) template was designed to convert the GGC codon found in exon 7 of the *Lat* gene and corresponding to the glycine residue present at position 135 of LAT into a GAC codon coding for an aspartic acid. The 110 nucleotides-long *Lat* ^G135D^ HDR template (5’-CAGAGCCAGCCTGTAAGAATGTGGATGCAGATGAGGATGAAGACGACTATCCCAACGACTACCTGTGAGTGGGTAGAGGGGAGGTGACCGTGGAAGTTGTGTGCCCTTTA-3’, the mutated basis is underlined) was synthesized as a ssDNA and purified using polyacrylamide gel electrophoresis (BiOligo, Shanghai, China). Fertilized eggs from C57BL/6 female mice were microinjected with Cas9 mRNA and the designed sgRNA and HDR template as described (13). Tail genomic DNA was isolated from the resulting F0 mice and a 386-bp region encompassing exon 7 amplified with the following pairs of PCR primers: 5’- GGCTTTGGGGAGGATGTACAA -3’ (Fwd) and 5’- CTCCAAGGTCAGGGTTGCTAG -3’ (Rev). Amplified products were then sequenced. An F0 mouse expressing the intended G135D mutation was used to established mice homozygous for the mutation. Mice were screened for the presence of the intended mutation by PCR with the oligonucleotides described above (Fwd and Rev), and the purified 386 bp PCR product was sequenced to discriminate between wild-type, heterozygous, or homozygous mutant animals.

### Preparation of cell lysates and western blotting

Thymocytes from wild-type or mutant mice were obtained and incubated with 5 µg/ml of biotin-coupled anti-CD3 mAb for 30 min on ice, and then stimulated at 37°C with 10 µg/ml of streptavidin. Cells were then lysed at 5.0 x 10^7^ cells/ml in 2X Laemmli buffer, followed by incubation at 95°C for 5 min and sonication. For Western blotting, whole-cell lysates were separated by SDS-PAGE and transferred to PVDF membranes, which were incubated with the indicated primary antibodies, followed by the appropriate secondary antibody conjugated to IRDye 800CW (Li-Cor, Lincoln, NE, USA) or horseradish peroxidase (HRP). Reactive proteins were visualized using the Odyssey CLx Infrared Imaging System (Li-Cor) or by enhanced chemiluminescence (ECL) acquired in a ChemiDoc Touch Imaging System (Bio-Rad Laboratories). For reprobing, PVDF membranes were incubated for 10 minutes at room temperature with WB Stripping Solution (Nacalai Tesque, Kyoto, Japan), followed by a TTBS wash. Western blots were densitometrically quantified, and statistics were performed with Microsoft Excel using a two-tailed t-test.

### Activation and proliferation assays

Splenocytes were obtained, and T lymphocytes were purified by negative selection MojoSort™ Mouse CD3 T Cell Isolation Kit (Biolegend, San Diego, San Diego, CA, USA). Purified T cells were then stained with CellTrace Violet™ for 20 min at 37°C. After staining, cells were collected and resuspended at 3.0 x 10^6^ cells/ml in RPMI1640 medium containing 10% Fetal Bovine Serum, sodium pyruvate 1 mM, β-mercaptoethanol 50 μM, and Penicillin-Streptomycin. Cells were dispatched in a 48 wells plate (1 x 10^6^ cells/well), and stimulated with different ratios of anti-CD3/CD28 coupled micro-beads: 8 x 10^4^ beads (ratio bead:cells = 1:12.5), 2 x 10^5^ beads (ratio = 1: 5), and 4 x 10^5^ beads (ratio = 1:2.5). Wells of unstimulated cells were also added as a control. 24 hours later, 100 μl of cells were recovered to analyze their activation by Flow Cytometry, after staining with specific antibodies for CD4, CD8 and CD69; only viable cells were analyzed (Annexin-V negative). At 72 hours, CD4 and CD8 T cell proliferation was analyzed by flow cytometry in the live cell population only.

### Ca^2+^ mobilization

Measurement of intracellular free Ca^2+^ in total thymocytes was carried out using Indo-1 AM (acetoxymethyl) (1 μM; Molecular Probes, Invitrogen) as previously described (14). Thymocytes were incubated with 5 µg/ml of biotin-conjugated anti-CD3 for 30 min on ice, and then stimulated with 10 µg/ml of streptavidin. Calcium measurements were performed using a Synergy MX Multi-Mode Reader (Biotek) at 37°C. Cells were excited by light at a wavelength of 340 nm, and the fluorescence emitted at 405 and 485 nm was collected alternately per second. Calcium mobilization was evaluated by the ratio of 405/485 nm fluorescence signal.

## Statistics

Statistics were performed with Microsoft Excel using a two-tailed t-test. Levels of significance p < 0.05 are presented as *, and p < 0.01 as **.

## Results and discussion

### LAT^G135D^ KI mouse generation

To address *in vivo* the significance of the residue preceding the sixth tyrosine found at position 136 of mouse LAT, we generated KI mice with a mutation that replaced the glycine residue found at position 135 with an aspartate residue (G135D). It has been previously demonstrated that the LAT^G135D^ mutation is able to increase and accelerate phosphorylation of the neighboring LAT-Y136 tyrosine residue (12, 15). LAT^G135D^ KI mice were generated using a CRISPR/Cas9 nuclease-based approach on a C57BL/6 background (see Material and Methods). Mice homozygous for this mutation LAT^G135D/G135D^ were born at expected Mendelian frequencies, and LAT^G135D/G135D^ T cells showed levels of LAT comparable to wild-type T cells (**Supplementary Figure 1A**).

### Altered thymic development in LAT^G135D^ mutant mice

**Figure 1:**
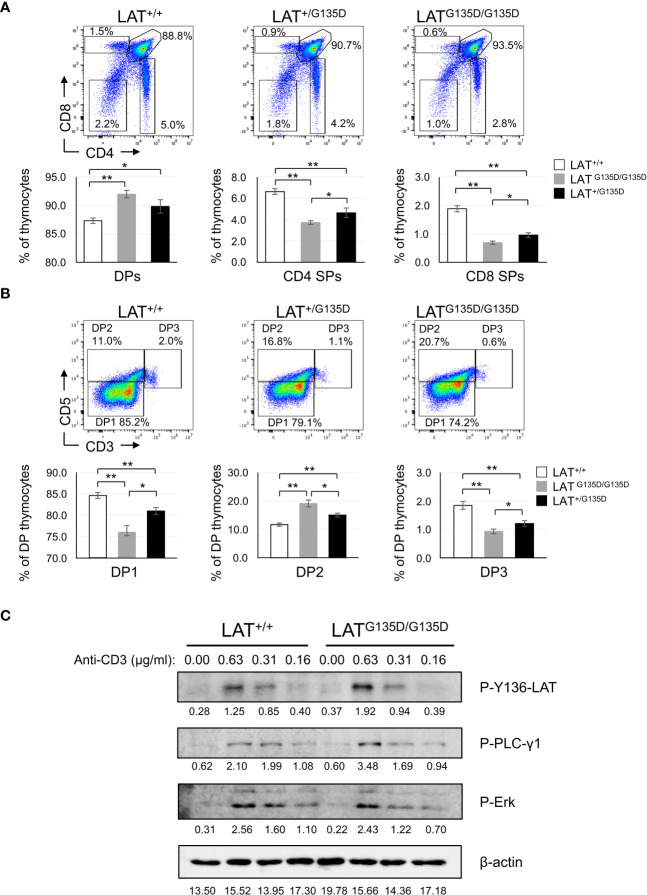
Thymic development analysis of LAT^G135D^ mice. **(A)** Total thymocytes from wild-type (LAT^+/+^), heterozygous (LAT^+/G135D^) and homozygous (LAT^G135D/G135D^) mutant mice were analyzed for the expression of CD4 and CD8 (upper panels) by flow cytometry. The numbers indicated in each gate represent the percentage of cells. Upper panels show a representative experiment. Lower panels represent bar graphs obtained after the analysis of LAT^+/+^ (n = 15), LAT^+/G135D^ (n = 9) and LAT^G135D/G135D^ (n = 13) mice, showing the percentages of cells in each of the indicated compartments. The brackets on each bar represent the mean standard error. **(B)** Dot plots showing CD5 and CD3 expression in DP cells from 6 to 14 weeks old wild-type (LAT^+/+^, n = 13), heterozygous (LAT^+/G135D^, n = 8) and homozygous (LAT^G135D/G135D^, n = 12) mice. Lower panels represent the quantification of percentages of preselection DP cells (DP1), DP cells undergoing selection (DP2), and post selection DP thymocytes (DP3). * indicates p<0.05; ** indicates p<0.01. **(C)** Analysis of TCR signaling in thymocytes. 5 x 10^6^ fresh thymocytes were obtained from wild-type (LAT^+/+^) and homozygous (LAT^G135D/G135D^) mutant mice and incubated with the indicated concentrations of biotin-conjugated anti-CD3 for 30 min, and then stimulated with 10 µg/ml of streptavidin for 3 min at 37°C. Cells were then lysed and analyzed by Western blot with the indicated specific antibodies. Membranes were stripped and reanalyzed with antibodies β-actin to show total protein load. Numbers below the images represent the densitometric quantification of each band.

We examined the influence of the G135D mutation on thymic development. The cellularity of the thymuses of heterozygous and homozygous LAT^G135D^ mutant mice showed no significant differences as compared to WT thymuses (**Supplementary Figure 1B**). Analysis of CD4 and CD8 expression showed an increase in the percentage of CD4+CD8+ double-positive (DP) compartment in both homozygous and heterozygous LAT^G135D^ mice as compared to wild-type mice. Conversely, the percentages of CD4+ and CD8+ single positive (SP) thymic populations were decreased in LAT^G135D^ mutant mice relative to wild-type (**Figure 1A**). To our knowledge, this constitutes the first heterozygous mutation in LAT affecting thymic development. LAT^G135D^ molecules are thus capable of exerting a trans-dominant effect in the presence of wild-type LAT molecules. The effect of positive selection can be monitored by analyzing the co-expression of CD5 and CD3 (1). The percentage of CD5^high^CD3^high^ (DP3) post-selection cells was reduced in both homozygous and heterozygous LAT^G135D^ mutant mice (**Figure 1B**). Likewise, the percentage of CD5^high^CD3^low/med^ (DP2) was increased in LAT^G135D^ mutant mice as compared to wild-type mice. Interestingly, the percentages of CD5^low^CD3^low/med^ (DP1), DP2 and DP3 populations found in heterozygous LAT^+/G135D^ mice were intermediate between those of the wild-type and homozygous mutant mice, showing statistically significant differences with both populations (**Figure 1B**). High CD5 expression levels on naive T cells have been correlated with high TCR self-reactivity (16), and CD4+ SP and CD8+ SP cells of LAT^+/G135D^ and LAT^G135D/G135D^ mutant mice expressed higher levels of CD5 as compared to wild-type mice (**Supplementary Figure 1D**), a result consistent with the view that LAT^G135D^ molecules deliver stronger TCR signals. Four distinct subsets of CD4-CD8- double-negative (DN) cells that can be defined using the expression of CD44 and CD25 showed no difference between wild-type and LAT mutant mice (**Supplementary Figure 1C**), suggesting that the LAT^G135D^ mutation does not affect the earliest stage of thymic development.

To gain further insights into the effects of the LAT^G135D^ mutation, we analyzed SP thymocyte maturation. SP successfully undergoing positive selection proceed through several stages before becoming fully mature SP T cells that acquire the competence to egress from the thymus. Down-regulation of CD24 (HSA) and up-regulation of CD62L have been used to characterize SP cell maturation and defines two distinct populations, corresponding to immature CD24^high^CD62L^med^, and more mature CD24^med^CD62L^high^ SP cells (17, 18). Consistent with the reduction in the percentage of the CD8+ SP cells, LAT^G135D/G135D^ homozygous mice showed a reduction in the mature CD8+CD24^med^CD62L^high^ population, and a concomitant increase in the CD8+CD24^high^CD62L^med^ immature population (**Supplementary Figure 2A**). The CD8+CD24^med^CD62L^high^ population was also diminished in LAT^G135D/G135D^ homozygous mice (**Supplementary Figure 2A**). Moreover, analysis of the expression level of CD3 in CD8+ SP cells showed an increase in the percentage of CD3^low^ cells and a corresponding decrease in the percentage of CD3^high^ cells, in both LAT^G135D/G135D^ and LAT^+/G135D^ mice as compared to wild-type mice (**Supplementary Figure 2B**). CD4+ SP thymocyte population showed the same trend, although in this case the differences are not so sharp (**Supplementary Figure 2B**). Altogether these data show that the LAT^G135D^ mutation impacts on the late stages of thymic development.

### Impact of the LAT^G135D^ mutation on TCR-CD3 signaling in thymocytes

We next sought to assess the impact of LAT^G135D^ mutation on TCR-CD3 signaling in thymocytes. Therefore, we analyzed intracellular signaling in thymocytes of LAT^G135D^ heterozygous and homozygous mutant mice after CD3 crosslinking and compared it to wild-type thymocytes. Fresh thymocytes were incubated on ice with biotin-coupled anti-CD3 antibodies at 5 μg/ml for 30 min, and then stimulated at 37°C with streptavidin for 0, 3, and 10 minutes. Cell lysates were prepared and analyzed by Western blotting. Anti-CD3 stimulation induced in wild-type thymocytes a strong increase in the phosphorylation of tyrosine 136 of LAT at 3 min, which was almost totally lost after 10 minutes (**Supplementary Figure 2A**). Similar results were obtained in heterozygous LAT^+/G135D^ and homozygous LAT^G135D/G135D^ mutant mice. Densitometric quantification of seven independent experiments showed no difference in the phosphorylation level of LAT-Y136 in mutant mice relative to wild-type mice (**Supplementary Figure 2B**). We next analyzed the effect of the G135D mutation on PLC-γ1 phosphorylation and Erk activation. As observed for LAT phosphorylation, CD3 cross-linking led to a rapid PLC-γ1 and Erk phosphorylation (**Supplementary Figure 2A**), and densitometric analysis showed no difference in the kinetics and intensities of phosphorylation of both proteins in the three types of mice (**Supplementary Figure 2B**). These results are somewhat puzzling, given the phenotypic differences observed in thymic development. Since the concentration of anti-CD3 used for stimulation might be too high and thus could mask subtle differences in intracellular signaling, stimulations were performed with 1/8, 1/16 and 1/32 dilutions of the initial concentration (i.e. 0.62, 0.31 and 0.15 μg/ml) for 3 minutes. As seen in **Figure 1C**, when thymocytes are stimulated with a concentration of 0.62 µg/ml the phosphorylation of PLC-γ1 and LAT tyrosine 136 are increased in mutant mice relative to wild type. This supports previous data generated by our group and others in Jurkat cells (12, 15). However, Erk phosphorylation (**Figure 1C**) and Ca^2+^ influx generation (**Supplementary Figure 3C**) do not appear to be increased in thymocytes from LAT-G135D mice. More work is needed to definitively elucidate these issues. Breeding these animals with mice containing a transgene for the TCR could definitively clarify whether the intensity of intracellular signals is increased and/or accelerated.

### Impact of the LAT^G135D^ mutation on peripheral lymphoid populations

**Figure 2:**
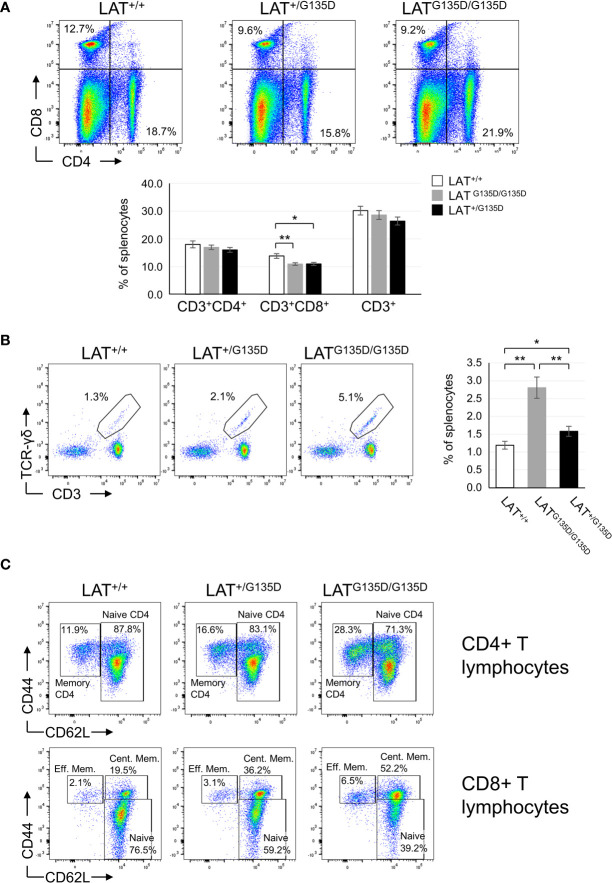
Peripheral lymphocyte populations are altered by the LAT-G135D mutation. **(A)** CD4 and CD8 expression was analyzed in splenocytes from wild-type (LAT^+/+^), heterozygous (LAT^+/G135D^), and homozygous (LAT^G135D/G135D^) mutant mice (upper panels) by flow cytometry. The numbers indicated in each quadrant represent the percentage of cells. Upper panels show a representative experiment. Lower bar graph represents the average of the indicated populations after the analysis of LAT^+/+^ (n = 14), LAT^+/G135D^ (n = 9), and LAT^G135D/G135D^ (n = 13) mice. The brackets on each bar represent the mean standard error. **(B)** Dot plots showing CD3 and TCR-γδ expression in splenocytes cells from the indicated types of mice. The bar graph on the right shows the percentages of γδ-T cells in each mouse type: n = 13 for LAT^+/+^, n = 9 for LAT^+/G135D^, and n = 11 for LAT^G135D/G135D^. **(C)** Analysis of memory and naive CD4 (upper dot plots) and CD8 (lower dot plots) T cells in spleen from wild-type (LAT^+/+^), heterozygous (LAT^+/G135D^), and homozygous (LAT^G135D/G135D^) mutant mice. The numbers in the depicted regions represent the percentage of cells.

We analyzed next the peripheral populations found in heterozygous LAT^+/G135D^ and homozygous LAT ^G135D/G135D^ mice. Although the total number of splenocytes did not differ significantly between wild-type and both heterozygous and homozygous mutant mice (**Supplementary Figure 1B**), the spleen showed a significant decrease in CD8+ T cells (**Figure 2A** and **Supplementary Figure 5A**), which was also found in lymph nodes (data not shown). Neither the percentage or number of CD4+ T cells, nor the percentage of total CD3+ T cells (**Figure 2A** and **Supplementary Figure 5A**) found in homozygous and heterozygous LAT mutant mice differed from wild-type mice and the same was true for lymph nodes (data not shown). Although the spleens of LAT^Y136F/Y136F^ mice showed increased numbers of B cells relative to their wild-type counterparts (7, 8), the heterozygous and homozygous LAT^G135D^ mutation had no detectable impact on the percentage of B lymphocytes found in the spleen (**Supplementary Figure 3A** and **Supplementary Figure 5A**) and in lymph node (not shown).

Interestingly, the numbers of γδ-T cells were increased in heterozygous LAT^+/G135D^ (1.6-fold) and homozygous (2.3-fold) LAT ^G135D/G135D^ mice, relative to wild-type animals (**Figure 2B** and **Supplementary Figure 5A**). It has been previously reported that a triple mutation of the three C-terminal tyrosines of LAT (LAT^3YF^ mice) triggers an expansion of γδ T cells (19). Therefore, the observed expansion of the γδ-T cell population of LAT^G135D^ mice could be related to an altered kinetics of phosphorylation of Tyr136. Despite the differences in both strains of mice, it is striking that allowing only tyrosine 136 phosphorylation (LAT^3YF^ mice), or favoring its phosphorylation (LAT^G135D^ mice), both result in an increase in the percentage of the γδ-T cells. As far as we know with the mice analyzed so far, no increase of the γδ-T cell population with age is observed. Three individuals older than 25 weeks have been analyzed and the percentage never reached 5% (not shown). Therefore, this phenotype is completely different from that observed in LAT-3YF mice (19), in which from 20 weeks onwards the T-gamma-delta cell population was huge.

Given that LAT is also expressed in NK cells (20, 21), we analyzed whether the LAT^G135D^ mutation had an impact on this cell compartment. As shown in **Supplementary Figure 4B**, there were not statistically significant differences in the NK1.1+DX5+ compartment among LAT^G135D^ mutant and wild-type splenocytes. However, a small increase in the NK1.1-DX5+ was observed in the spleens of homozygous LAT^G135D/G135D^ mutant mice relative to wild-type animals (**Supplementary Figure 3B**, lower panel). Although the increase is very small (2.99 ± 0.14 in LAT^+/+^ and 3.63 ± 0.14 in LAT^G135D/G135D^ mice), this difference reaches statistical significance. However, this NK1.1 negative DX5 positive population cannot be categorized as true NK cells (22). Considering that DX5 antigen – also known as CD49b or as α2 integrin – has been also identified as a memory differentiation marker of T cells (23), we analyzed whether this small increase in the NK1.1-DX5+ population is due to an increase in the percentage of memory T cells.

### Study of the naive and memory T cell compartments present in LAT^G135D^ mice

The CD44+CD62L- memory population of splenic CD4+ T cells showed augmented percentage in homozygous LAT^G135D/G135D^ mice relative to wild-type mice, and heterozygous LAT^+/G135D^ mice showed percentages of memory CD44+CD62L- cells that were intermediate between LAT^G135D/G135D^ mice and wild-type mice (**Figure 2C**). However, these differences constituted a trend and did not reach statistical significance (**Supplementary Figure 4C**). In contrast, the analysis of splenic CD8+ T cells showed a striking increase in the percentage of CD44+CD62L+ central memory cells in both heterozygous LAT^+/G135D^ and homozygous LAT^G135D/G135D^ mice relative to wild-type animals (**Figure 2C** and **Supplementary Figure 4C**). As a result, the percentage of naive CD8+ T cells (CD44^low/neg^CD62L+) was decreased in mutant mice. Similar results were also observed in lymph nodes (**Supplementary Figure 4C**), as well as when the comparison of absolute numbers of CD8+ T cells was performed (**Supplementary Figure 5B**). The percentages of central memory CD8 T cells of heterozygous LAT^+/G135D^ mice were intermediate between those of homozygous LAT^G135D/G135D^ and wild-type mice (**Supplementary Figure 4C**), showing statistically significant differences among the three types of mice. The CD44+CD62L- effector memory subset of splenic CD8 T cells showed no significant difference between wild-type and G135D (homozygous or heterozygous) mutant mice. Therefore, the LAT^G135D^ mutation favors the differentiation of naive CD8+ T cells into central memory cells. These cells have a high proliferative capacity after reencountering specific antigen, and they also show self-renewal ability (24). Therefore, the LAT^G135D^ mutation affects CD8+ T lymphocytes to a greater extent than CD4+ T cells, despite the fact that both CD4+ and CD8+ SP thymocyte populations are affected.

### Anergic T cells phenotype in LAT^G135D^ mutant mice

**Figure 3:**
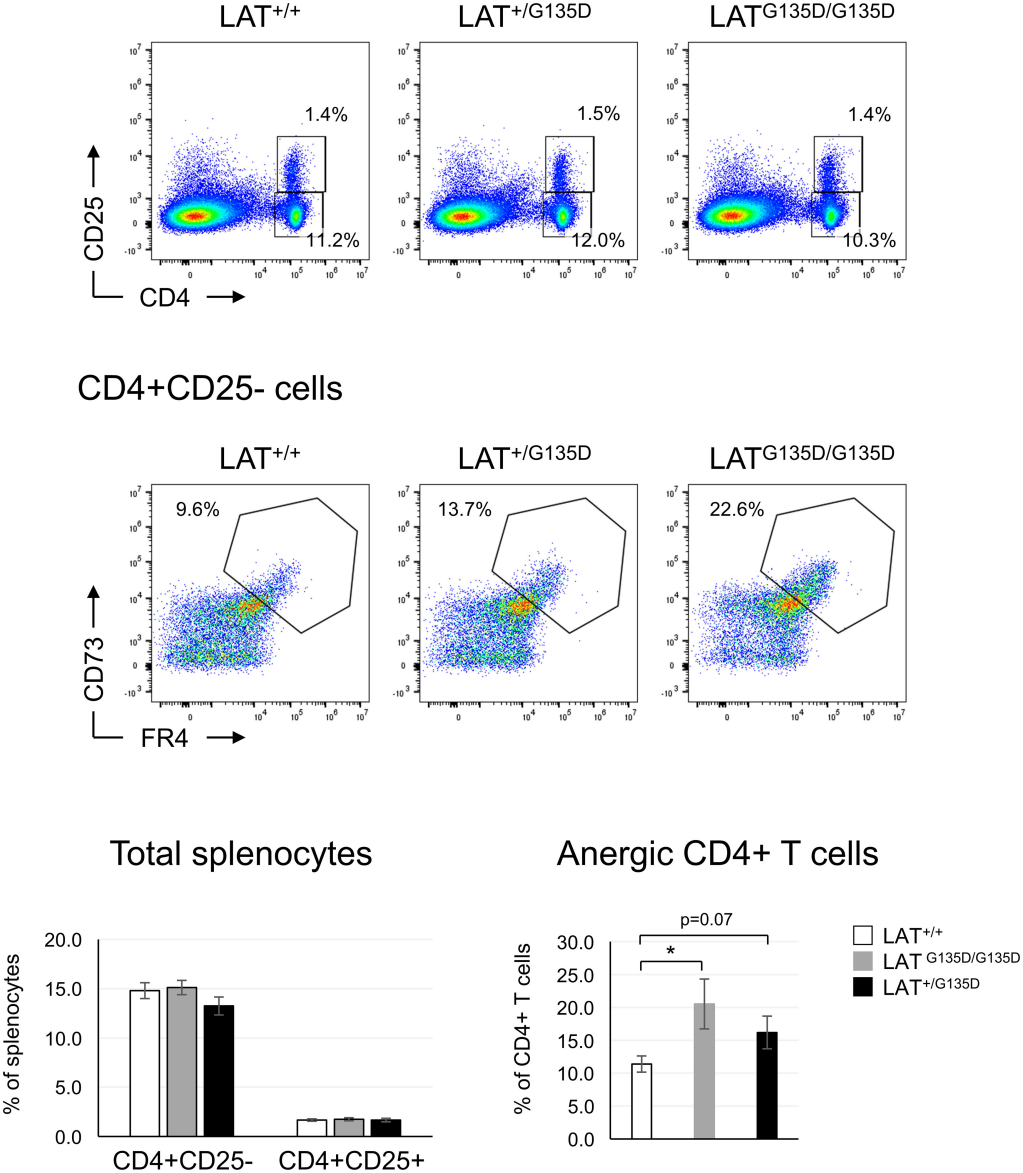
LAT^G135D^ mutation increases the percentage of anergic T cells. Splenocytes from the indicated types of mice were stained with CD4 and CD25 specific monoclonal antibodies (upper dot plots). The numbers indicated in each gate represent the percentage of CD4^+^CD25^-^ and CD4^+^CD25^+^ cells. Middle panels: CD4^+^CD25^-^ cells were analyzed for the expression of CD73 and FR4, and the percentage of anergic T cells is indicated. Lower bar graphs: average of the indicated populations after the analysis of LAT^+/+^ (n = 13), LAT^+/G135D^ (n = 8) and LAT^G135D/G135D^ (n = 12) mice. The brackets on each bar represent the mean standard error. * indicates p<0.05.

It has been previously shown that proper integration of calcium signals with other signaling pathways results in full T cell activation, while unopposed calcium signaling leads to anergy (25, 26). Although we have not been able to demonstrate an increase in calcium influxes, previous data obtained in Jurkat cells show that the LAT^G135D^ mutation potentiates PLC-γ1 activation and Ca^2+^ responses (12, 15). Therefore, it was of interest to analyze whether the LAT^G135D^ mutation increased the percentage of anergic T cells. We have previously reported that J.CaM2 cells expressing the LAT^G131D^ mutant are more sensitive to inhibition of IL-2 production by pre-treatment with anti-CD3, which points to a possible role of this residue in the generation of anergy (15). Therefore, we assessed the presence of anergic T cells in spleens from wild-type, LAT^+/G135D^ heterozygous, and LAT^G135D/G135D^ homozygous mice. CD73 and Folate Receptor 4 (FR4) constitute reliable surface markers of anergic CD4+ T cells (27), and splenic conventional CD4+CD25- T cells were thus stained with specific antibodies for both markers. Analysis of the CD73^high^FR4^high^ population demonstrated an 1.8-fold increase in anergic T cells in mutant mice, with a statistically significant difference between homozygous and wild-type mice (**Figure 3**). These CD73^high^ FR4^high^ cells, in contrast to non-anergic CD4+ cells, were unable to proliferate in response to CD3/CD28 stimulation, supporting their anergic phenotype (**Supplementary Figure 5C**). Therefore, these results support our previous report (15) suggesting that the LAT^G135D^ mutation promotes entry into anergy of CD4+ T cells. Note that similar percentages of CD4+CD25+FoxP3+ cells were present in the spleens of wild-type and mutant mice (**Figure 3** and **Supplementary Figure 4C**), suggesting that development of Treg cells was not affected by the LAT^G135D^ mutation.

### Increased activation and proliferation of LAT^G135D/G135D^ homozygous mutant T cells

**Figure 4:**
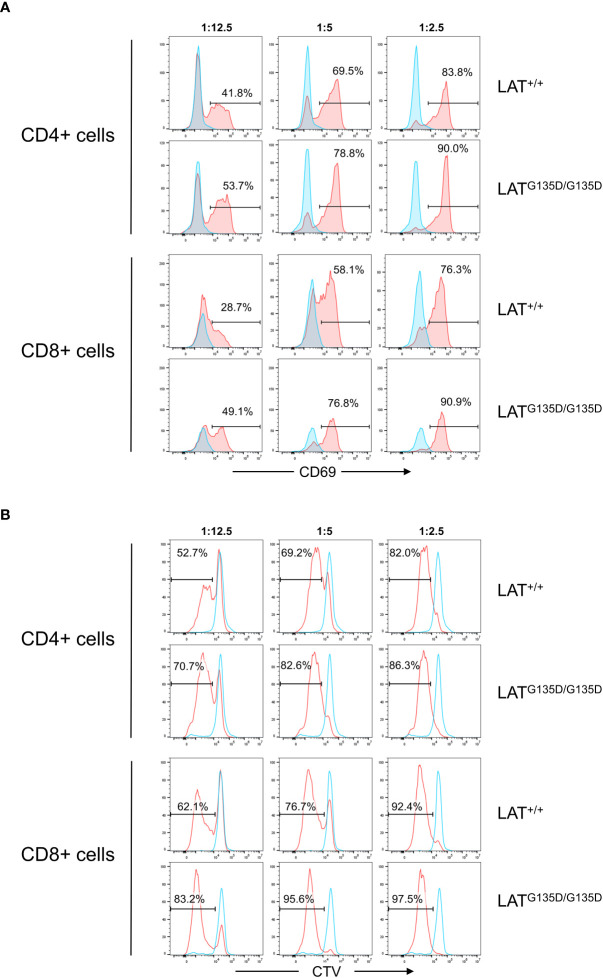
Ex vivo stimulation assays. **(A)** T cells purified from the spleens of wild-type LAT (LAT^+/+^) and homozygous (LAT^G135D/G135D^) mutant mice were stimulated with the indicated bead:cell ratios of anti-CD3/CD28 microbeads, and analysis of CD69 surface expression 24H post-stimulation was performed on live cells (Annexin V negative). CD69 expression in CD4+ and CD8+ cell populations are shown separately. The numbers indicated in each histogram represent the percentage of CD69 positive cells (red histograms). Blue histograms show CD69 expression in unstimulated cells. One representative experiment is shown (n=3). **(B)** Analysis of proliferation performed at 72H post-stimulation. Decrease of CTV staining indicates cell proliferation. Blue histograms correspond to unstimulated cells and red histograms to anti-CD3/CD28 stimulated cells. Proliferative capacity was analyzed separately in CD4+ and CD8+ cell populations. The numbers shown in each histogram represent the percentage of cells that have proliferated. One representative experiment is shown (n=3).

Finally, we sought to assess whether the LAT^G135D^ mutation favors activation and proliferation of mature T lymphocytes. Thus, we negatively purified CD3^+^ T cells from the spleens of wild-type and homozygous LAT^G135D/G135D^ mutant mice. Purified CD3^+^ T cells comprised greater than 95%, and contained lower percentages of CD8+ T cells in homozygous mutant mice (not shown). Purified cells were labeled with Cell Trace Violet (CTV) dye in order to test their proliferative capacity following stimulation with anti-CD3/CD28 antibodies coupled micro-beads, as previously described (28). Purified and CTV-labeled T cells were stimulated at 37°C with three different CD3/CD28 micro-beads to cells ratios, and analyzed for the expression of the CD69 activation marker after 24 hours of stimulation. Cells were simultaneously stained with Annexin V to discard dead cells, and for CD4 and CD8 to separately analyze each of these populations. **Figure 4A** shows that both CD4+ and CD8+ T cells from LAT^G135D/G135D^ mutant mice show increased percentages of CD69^+^ cells relative to wild-type. The increase in CD69 expression is greater at lower cell:bead ratios, and it also appears that the increase is somewhat greater in CD8+ cells than in CD4+ cells. Consistent with these results, the proliferative capacity of both CD4^+^ and CD8^+^ T cells from mutant mice was increased relative to those from wild-type mice (**Figure 4B**). Again, the differences were greater at low cell:bead ratios. These results support an increased signaling capability of LAT^G135D^ mutant in mature T lymphocytes.

In conclusion, our results involving the analysis of the phenotype of LAT^G135D^ mutant mice suggested that this mutation increased positive and negative selection during thymic development, and this occurred even in heterozygous LAT^G135D^ mice. The LAT^G135D^ mutation has thus a dominant or co-dominant character, since heterozygous LAT^G135D^ mice exhibit clear differences with wild-type mice in thymic and peripheral phenotypes. To our knowledge, this is the first time that a mutation in the LAT adaptor capable of modifying the development or activation of T cells in a heterozygous state. Our preliminary results suggest that favoring phosphorylation of LAT-Y136 increases the autoimmune potential of T cells, although more work is needed to identify the molecular mechanisms involved on that phenomenon. Interestingly, the LAT^G135D^ mutation, which increases phosphorylation of LAT-Y136, affects CD8+ T cells function more than that of CD4+ cells, and this markedly contrasts with the LAT^Y136F^ mutation (7, 8). Along that line, it would be of interest to develop mice harboring compound LAT^Y136F^ and LAT^G135D^ mutations and analyze the resulting phenotype. The increased impact of the LAT^G135D^ mutation on peripheral CD8+ T cells is intriguing, given that both CD4+ and CD8+ SP thymocyte populations are affected. A potential explanation for this discrepancy may be that numbers of mature CD4+ and CD8+ T lymphocyte subpopulations are independently regulated, and that the LAT^G135D^ mutation increases tonic signaling, which could affect differently to both subpopulations. Indeed, homeostatic proliferation and apoptosis are different for CD4+ and CD8+ peripheral lymphocytes (29). Further studies are needed to identify the intracellular signals involved in the differential phenotype of peripheral CD4+ and CD8+ lymphocytes.

## Data availability statement

The original contributions presented in the study are included in the article/**Supplementary Material**. Further inquiries can be directed to the corresponding author.

## Ethics statement

The animal study was reviewed and approved by Animal Care and Ethics Committee (University of Cadiz, Cadiz, Spain) and the Ministry of Agriculture, Fisheries and Rural Development (Junta de Andalucía, Spain).

## Author contributions

MMA-E, IV-B, and FZ performed most of the experiments and interpreted results. LMF-A, MC, and IN-S helped with cell culture, animal handling and genotyping, and signaling experiments. MMA-E, IV-B, and FZ provided input on study design. LZ and YL generated the LAT^G135D^ mice. FZ and BM contributed to the establishment and phenotyping of the LAT^G135D^ mice. EA wrote the manuscript and directed the study. All authors contributed to the article and approved the submitted version.

## Supplementary material

Supplementary Figure 1 Effects of the LAT-G135D mutation on thymic development and cellularity of lymphoid organs. **(A)** Western blot analysis of LAT proteins in thymocytes isolated from wild-type (LAT^+/+^), heterozygous (LAT^+/G135D^) and homozygous (LAT^G135D/G135D^) mutant mice. Cell lysates were analyzed by Western blot using an anti-LAT rabbit mAb. The blot was stripped and reblotted for β-actin. **(B)** Quantification of total cell number in thymus and spleen of wild-type (LAT^+/+^, n = 15), heterozygous (LAT^+/G135D^, n = 9) and homozygous (LAT^G135D/G135D^, n = 13) mutant mice. **(C)** DN thymocytes (gated on CD3- CD4- CD8- and B220- cells) from 6 to 14 weeks old wild-type (LAT^+/+^, n = 12), heterozygous (LAT^+/G135D^, n = 9), and homozygous (LAT^G135D/G135D^, n = 7) mutant mice were analyzed for the expression of CD44 and CD25. Percentages of cells are shown in each quadrant. The lower bar graph represents the percentages of cells in each of the indicated compartments. Brackets on each bar represent the mean standard error. **(D)** CD5 expression in CD4+ SP (left histogram) and CD8+ SP thymocytes (right histogram) from wild-type (LAT^+/+^, blue line) and homozygous (LAT^G135D/G135D^, red line) mutant mice. The bar graph on the right shows the Mean Fluorescence Intensity (MFI, arbitrary units) in CD4+ SP and CD8 SP thymocytes from 6 to 14 weeks old wild-type (LAT^+/+^, n = 12), heterozygous (LAT^+/G135D^, n = 8), and homozygous (LAT^G135D/G135D^, n = 10) mutant mice. E) Quantification of cell numbers in thymi from wild-type (LAT^+/+^, n = 12), heterozygous (LAT^+/G135D^, n = 6) and homozygous (LAT^G135D/G135D^, n = 10) mutant mice. Brackets represent the standard error. * indicates p<0.05; ** indicates p<0.01.

Supplementary Figure 2 Terminal thymic development of SP cells in LAT^G135D^ mice. **(A)** Terminal SP thymic development was analyzed by the expression of CD62L and CD24 in CD4+ and CD8+ SP thymocytes (upper dot plots). The lower bar graphs represent the percentages of Immature and Mature SP cells from 6 to 14 weeks old wild-type (LAT^+/+^, n = 8), heterozygous (LAT^+/G135D^, n = 7) and homozygous (LAT^G135D/G135D^, n = 10) mutant mice. Brackets on each bar represent the mean standard error. **(B)** Analysis of CD3 expression in CD4+ SP and CD8+ SP thymocytes (upper dot plots). The lower bar graph represents the percentages of CD3^low^ and CD3^high^ populations in CD4+ or CD8+ SP cells from 6 to 14 weeks old wild-type (LAT^+/+^, n = 13), heterozygous (LAT^+/G135D^, n = 8) and homozygous (LAT^G135D/G135D^, n = 12) mutant mice. Brackets on each bar represent the mean standard error. * indicates p<0.05; ** indicates p<0.01.

Supplementary Figure 3 Analysis of TCR signaling in thymocytes. **(A)** 5 x 10^6^ fresh thymocytes were obtained from wild-type (LAT^+/+^), heterozygous (LAT^+/G135D^) and homozygous (LAT^G135D/G135D^) mutant mice and incubated with 5 µg/ml of biotin-conjugated anti-CD3 for 30 min, and then stimulated with 10 µg/ml of streptavidin for 0, 3 and 10 min at 37°C. Cells were then lysed and analyzed by Western blot with the indicated specific antibodies. Membranes were stripped and reanalyzed with antibodies against total LAT, PLC-γ1, and Erk to show total protein load. One representative experiment out of a total of seven is shown. **(B)** Quantification of TCR signaling in thymocytes from wild-type (LAT^+/+^), heterozygous (LAT^+/G135D^) and homozygous (LAT^G135D/G135D^) mutant mice. Densitometry was performed in seven independent experiments, and they represent the mean fold increase in phosphorylation of LAT-Tyr136, PLC-γ1 and Erk. Phosphorylation levels were normalized to total LAT, PLCγ1 or Erk expression. Brackets represent the standard error. **(C)** Thymocytes from wild-type (LAT^+/+^) and homozygous mutant mice (LAT^G135D/G135D^) were loaded with Indo-1AM and stimulated with 5 µg/ml of biotin-conjugated anti-CD3 for 30 min, and then stimulated with 10 µg/ml of streptavidin at the indicated time (black arrow). The intracellular Ca^2+^ concentration was determined at 37°C through the change in Indo-1AM fluorescence.

Supplementary Figure 4 Cell populations in the spleen. **(A)** Histograms showing the percentages of B cells of splenocytes from wild-type (LAT^+/+^), heterozygous (LAT^+/G135D^) and homozygous (LAT^G135D/G135D^) mutant mice. **(B)** Upper panels: dot plots showing NK cells populations analyzed by the expression of NK1.1 and DX5 markers. The numbers indicated in each quadrant represent the percentage of cells. Upper panels show a representative experiment. Lower bar graph represents the average of the indicated populations after the analysis of LAT^+/+^ (n = 12), LAT^+/G135D^ (n = 9) and LAT^G135D/G135D^ (n = 9) mice. The brackets on each bar represent the mean standard error. **(C)** Intracytoplasmic staining of FoxP3 of CD4+CD25+ T cells obtained from the spleens of wild-type (LAT^+/+^) and homozygous (LAT^G135D/G135D^) mutant mice. Gray shaded area represents the isotype control. **(C)** Bar graphs showing the indicated populations of naive and memory T cells in the spleens from wild-type (LAT^+/+^, n = 8), heterozygous (LAT^+/G135D^, n = 7) and homozygous (LAT^G135D/G135D^, n = 10) mutant mice. For lymph nodes, the data are from 7 LAT^+/+^, 4 LAT^+/G135D^, and 10 LAT^G135D/G135D^ mice.

Supplementary Figure 5 Splenic cellularity and functional analysis of anergic cells. **(A)** Quantification of cell numbers for the indicated lymphocytic populations from wild-type (LAT^+/+^, n = 12), heterozygous (LAT^+/G135D^, n = 6) and homozygous (LAT^G135D/G135D^, n = 10) mutant mice. **(B)** Bar graphs showing cell numbers of the indicated populations of naive and memory T cells in the spleens from wild-type (LAT^+/+^, n = 8), heterozygous (LAT^+/G135D^, n = 7) and homozygous (LAT^G135D/G135D^, n = 10) mutant mice. Brackets represent the standard error. * indicates p<0.05; ** indicates p<0.01. **(C)** Analysis of proliferation of non-anergic and anergic (CD73^high^ FR4^high^) CD4+ T cells performed at 72H post-stimulation after stimulation with anti-CD3/CD28 microbeads at a 1:2.5 bead:cell ratio. Decrease of CTV staining indicates cell proliferation. Blue histograms correspond to unstimulated cells and red histograms to anti-CD3/CD28 stimulated cells. The numbers shown in each histogram represent the percentage of cells that have proliferated.
