# Supplementary figures and images for: Mutation of the glycine residue preceding the sixth tyrosine of the LAT adaptor severely alters T cell development and activation
Source: Front Immunol. 2022 Dec 7;13:1054920. doi: 10.3389/fimmu.2022.1054920 (PMC9768323; doi:10.3389/fimmu.2022.1054920)

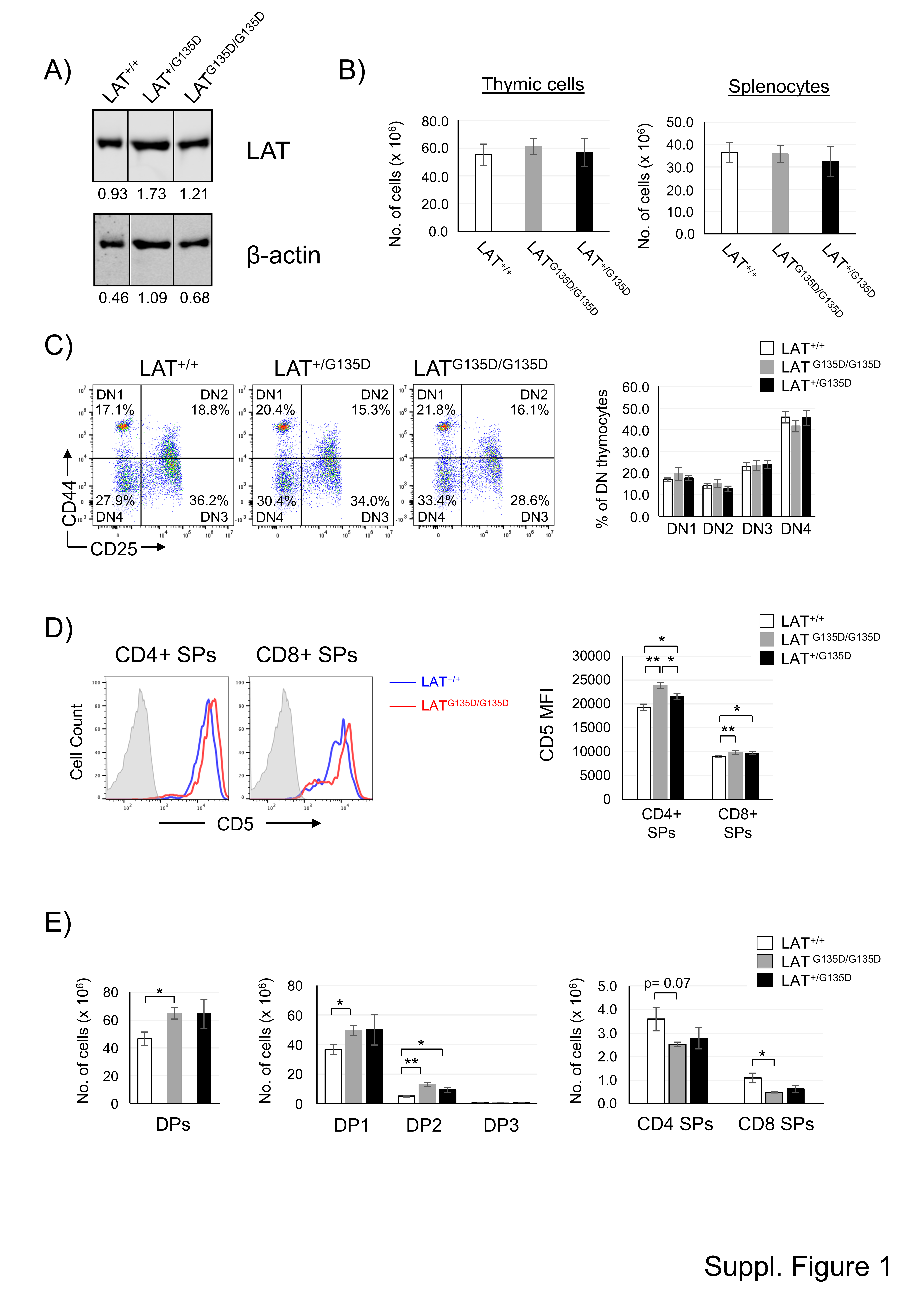

Supplement: Supplementary Figure 1 — Effects of the LAT-G135D mutation on thymic development and cellularity of lymphoid organs. (A) Western blot analysis of LAT proteins in thymocytes isolated from wild-type (LAT+/+), heterozygous (LAT+/G135D) and homozygous (LATG135D/G135D) mutant mice. Cell lysates were analyzed by Western blot using an anti-LAT rabbit mAb. The blot was stripped and reblotted for β-actin. (B) Quantification of total cell number in thymus and spleen of wild-type (LAT+/+, n = 15), heterozygous (LAT+/G135D, n = 9) and homozygous (LATG135D/G135D, n = 13) mutant mice. (C) DN thymocytes (gated on CD3- CD4- CD8- and B220- cells) from 6 to 14 weeks old wild-type (LAT+/+, n = 12), heterozygous (LAT+/G135D, n = 9), and homozygous (LATG135D/G135D, n = 7) mutant mice were analyzed for the expression of CD44 and CD25. Percentages of cells are shown in each quadrant. The lower bar graph represents the percentages of cells in each of the indicated compartments. Brackets on each bar represent the mean standard error. (D) CD5 expression in CD4+ SP (left histogram) and CD8+ SP thymocytes (right histogram) from wild-type (LAT+/+, blue line) and homozygous (LATG135D/G135D, red line) mutant mice. The bar graph on the right shows the Mean Fluorescence Intensity (MFI, arbitrary units) in CD4+ SP and CD8 SP thymocytes from 6 to 14 weeks old wild-type (LAT+/+, n = 12), heterozygous (LAT+/G135D, n = 8), and homozygous (LATG135D/G135D, n = 10) mutant mice. E) Quantification of cell numbers in thymi from wild-type (LAT+/+, n = 12), heterozygous (LAT+/G135D, n = 6) and homozygous (LATG135D/G135D, n = 10) mutant mice. Brackets represent the standard error. * indicates p<0.05; ** indicates p<0.01. [file Image_1.tif]

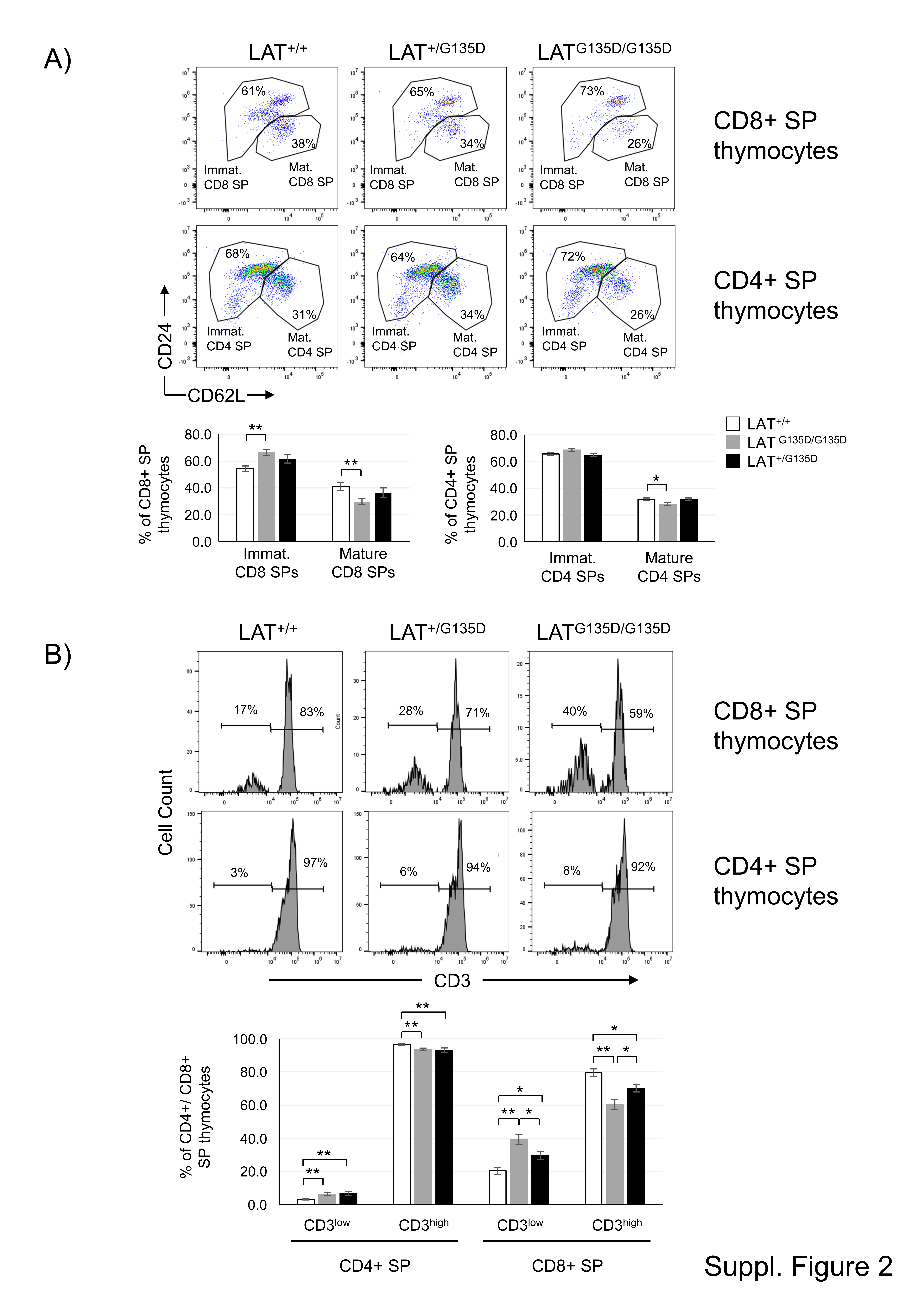

Supplement: Supplementary Figure 2 — Terminal thymic development of SP cells in LATG135D mice. (A) Terminal SP thymic development was analyzed by the expression of CD62L and CD24 in CD4+ and CD8+ SP thymocytes (upper dot plots). The lower bar graphs represent the percentages of Immature and Mature SP cells from 6 to 14 weeks old wild-type (LAT+/+, n = 8), heterozygous (LAT+/G135D, n = 7) and homozygous (LATG135D/G135D, n = 10) mutant mice. Brackets on each bar represent the mean standard error. (B) Analysis of CD3 expression in CD4+ SP and CD8+ SP thymocytes (upper dot plots). The lower bar graph represents the percentages of CD3low and CD3high populations in CD4+ or CD8+ SP cells from 6 to 14 weeks old wild-type (LAT+/+, n = 13), heterozygous (LAT+/G135D, n = 8) and homozygous (LATG135D/G135D, n = 12) mutant mice. Brackets on each bar represent the mean standard error. * indicates p<0.05; ** indicates p<0.01. [file Image_2.tif]

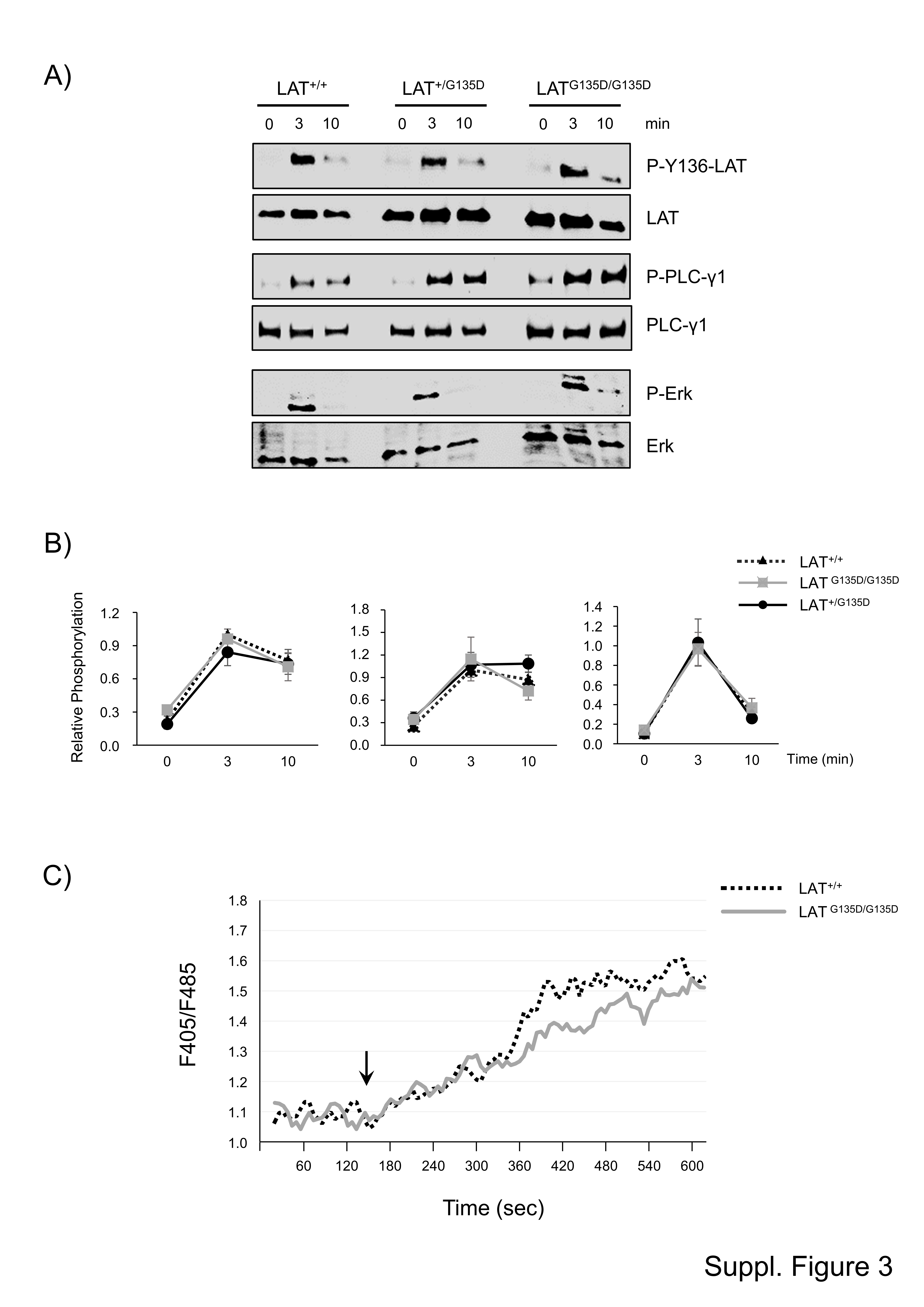

Supplement: Supplementary Figure 3 — Analysis of TCR signaling in thymocytes. (A) 5 x 10^6^ fresh thymocytes were obtained from wild-type (LAT+/+), heterozygous (LAT+/G135D) and homozygous (LATG135D/G135D) mutant mice and incubated with 5 µg/ml of biotin-conjugated anti-CD3 for 30 min, and then stimulated with 10 µg/ml of streptavidin for 0, 3 and 10 min at 37°C. Cells were then lysed and analyzed by Western blot with the indicated specific antibodies. Membranes were stripped and reanalyzed with antibodies against total LAT, PLC-γ1, and Erk to show total protein load. One representative experiment out of a total of seven is shown. (B) Quantification of TCR signaling in thymocytes from wild-type (LAT+/+), heterozygous (LAT+/G135D) and homozygous (LATG135D/G135D) mutant mice. Densitometry was performed in seven independent experiments, and they represent the mean fold increase in phosphorylation of LAT-Tyr136, PLC-γ1 and Erk. Phosphorylation levels were normalized to total LAT, PLCγ1 or Erk expression. Brackets represent the standard error. (C) Thymocytes from wild-type (LAT+/+) and homozygous mutant mice (LATG135D/G135D) were loaded with Indo-1AM and stimulated with 5 µg/ml of biotin-conjugated anti-CD3 for 30 min, and then stimulated with 10 µg/ml of streptavidin at the indicated time (black arrow). The intracellular Ca2+ concentration was determined at 37°C through the change in Indo-1AM fluorescence. [file Image_3.tif]

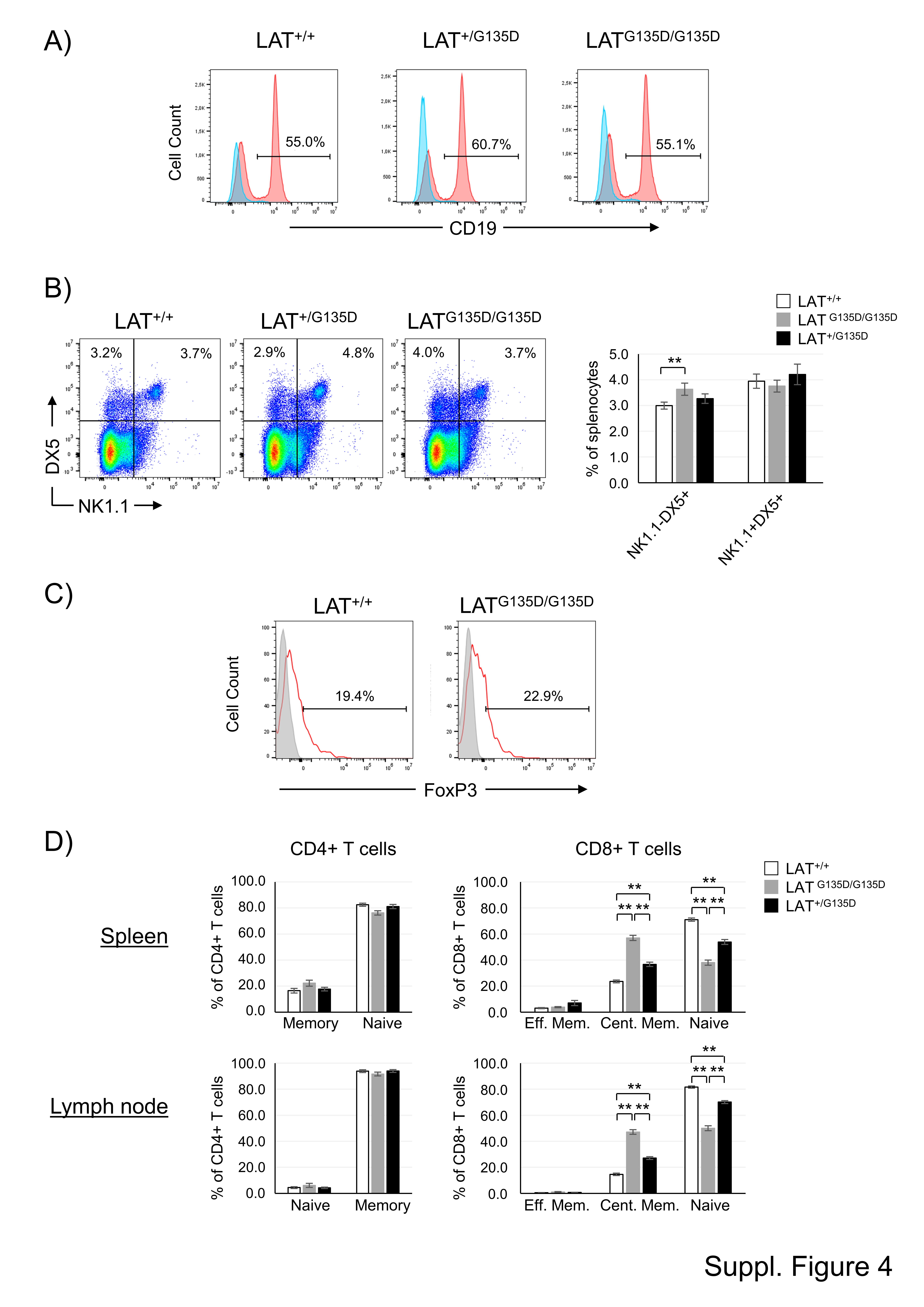

Supplement: Supplementary Figure 4 — Cell populations in the spleen. (A) Histograms showing the percentages of B cells of splenocytes from wild-type (LAT+/+), heterozygous (LAT+/G135D) and homozygous (LATG135D/G135D) mutant mice. (B) Upper panels: dot plots showing NK cells populations analyzed by the expression of NK1.1 and DX5 markers. The numbers indicated in each quadrant represent the percentage of cells. Upper panels show a representative experiment. Lower bar graph represents the average of the indicated populations after the analysis of LAT+/+ (n = 12), LAT+/G135D (n = 9) and LATG135D/G135D (n = 9) mice. The brackets on each bar represent the mean standard error. (C) Intracytoplasmic staining of FoxP3 of CD4+CD25+ T cells obtained from the spleens of wild-type (LAT+/+) and homozygous (LATG135D/G135D) mutant mice. Gray shaded area represents the isotype control. (C) Bar graphs showing the indicated populations of naive and memory T cells in the spleens from wild-type (LAT+/+, n = 8), heterozygous (LAT+/G135D, n = 7) and homozygous (LATG135D/G135D, n = 10) mutant mice. For lymph nodes, the data are from 7 LAT+/+, 4 LAT+/G135D, and 10 LATG135D/G135D mice. [file Image_4.tif]

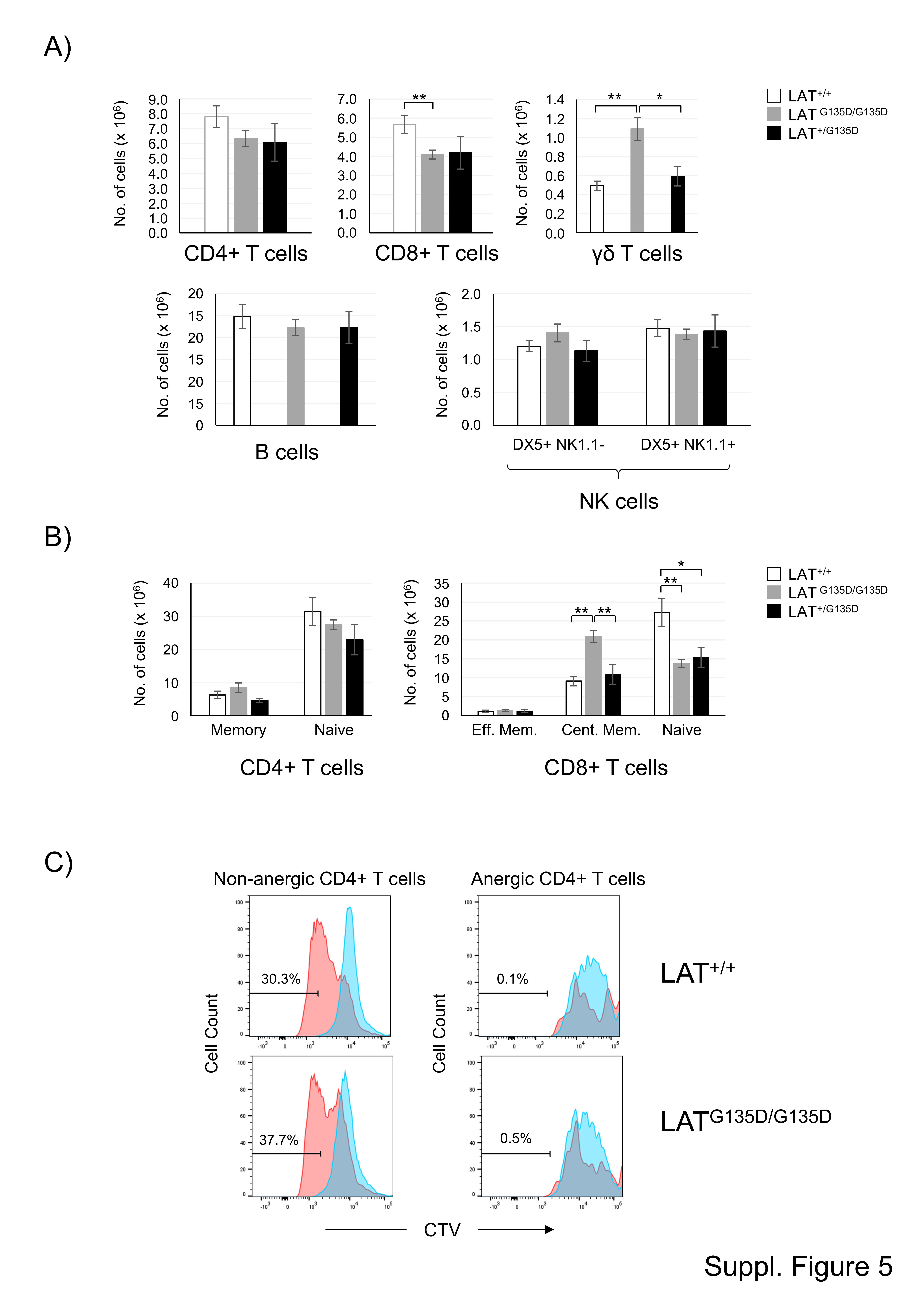

Supplement: Supplementary Figure 5 — Splenic cellularity and functional analysis of anergic cells. (A) Quantification of cell numbers for the indicated lymphocytic populations from wild-type (LAT+/+, n = 12), heterozygous (LAT+/G135D, n = 6) and homozygous (LATG135D/G135D, n = 10) mutant mice. (B) Bar graphs showing cell numbers of the indicated populations of naive and memory T cells in the spleens from wild-type (LAT+/+, n = 8), heterozygous (LAT+/G135D, n = 7) and homozygous (LATG135D/G135D, n = 10) mutant mice. Brackets represent the standard error. * indicates p<0.05; ** indicates p<0.01. (C) Analysis of proliferation of non-anergic and anergic (CD73high FR4high) CD4+ T cells performed at 72H post-stimulation after stimulation with anti-CD3/CD28 microbeads at a 1:2.5 bead:cell ratio. Decrease of CTV staining indicates cell proliferation. Blue histograms correspond to unstimulated cells and red histograms to anti-CD3/CD28 stimulated cells. The numbers shown in each histogram represent the percentage of cells that have proliferated. [file Image_5.tif]
